# Somaclonal Variation for Genetic Improvement of Starch Accumulation in Potato (*Solanum tuberosum*) Tubers

**DOI:** 10.3390/plants12020232

**Published:** 2023-01-04

**Authors:** Walaa M. R. M. Adly, Gniewko Niedbała, Mohammad E. EL-Denary, Mahasen A. Mohamed, Magdalena Piekutowska, Tomasz Wojciechowski, El-Sayed T. Abd El-Salam, Ahmed S. Fouad

**Affiliations:** 1 Horticulture Research Institute, Agriculture Research Center, Giza 12619, Egypt; 2 Department of Biosystems Engineering, Faculty of Environmental and Mechanical Engineering, Poznań University of Life Sciences, Wojska Polskiego 50, 60-627 Poznań, Poland; 3 Department of Geoecology and Geoinformation, Institute of Biology and Earth Sciences, Pomeranian University in Słupsk, Partyzantów 27, 76-200 Słupsk, Poland; 4 Botany and Microbiology Department, Faculty of Science, Cairo University, Giza 12613, Egypt

**Keywords:** potato, tissue culture, somaclonal variation, starch, gene expression

## Abstract

Starch content is one of the major quality criteria targeted by potato breeding programs. Traditional potato breeding is a laborious duty due to the tetraploid nature and immense heterozygosity of potato genomes. In addition, screening for functional genetic variations in wild relatives is slow and strenuous. Moreover, genetic diversity, which is the raw material for breeding programs, is limited due to vegetative propagation used in the potato industry. Somaclonal variation provides a time-efficient tool to breeders for obtaining genetic variability, which is essential for breeding programs, at a reasonable cost and independent of sophisticated technology. The present investigation aimed to create potato somaclones with an improved potential for starch accumulation. Based on the weight and starch content of tubers, the somaclonal variant *Ros 119*, among 105 callus-sourced clones, recorded a higher tuberization potential than the parent cv Lady Rosetta in a field experiment. Although this somaclone was similar to the parent in the number of tubers produced, it exhibited tubers with 42 and 61% higher fresh and dry weights, respectively. Additionally, this clone recorded 10 and 75% increases in starch content based on the dry weight and average content per plant, respectively. The enhanced starch accumulation was associated with the upregulation of six starch-synthesis-related genes, namely, the *AGPase, GBSS I, SBE I, SBE II, SS II* and *SS III* genes. AGPase affords the glycosyl moieties required for the synthesis of amylose and amylopectin. GBSS is required for amylose elongation, while SBE I, SBE II, SS II and SS III are responsible for amylopectin.

## 1. Introduction

Potato (*Solanum tuberosum*) is the most crucial non-graminaceous food crop cultivated in 17.34 million hectares yielding 370 million tons (FAO 2019 https://www.fao.org/faostat/ar/#data/QCL, accessed on 23 October 2022). Tubers are an important source for carbohydrates, proteins and other essential nutrients including minerals (iron, magnesium, phosphorus, potassium and zinc), vitamins (thiamin, niacin, pyridoxine, riboflavin, folate and ascorbic acid, pantothenic acid), and dietary fibers [1]. Potato stands first among food crops for production of energy, proteins, vitamins and minerals per unit of land area and time [2]. Alongside the indoor culinary purposes, tubers are utilized in many food products [3] that accumulate massive amounts of peel directed to bioethanol production [4].

Starch is the most abundant carbohydrate in potato tubers; it is a mixture of two polysaccharides. The first is amylose which is a linear, long α-glucan with few branches, containing about 99% α-(1,4) linkages and only 1% α-(1,6) linkages. The second is amylopectin characterized with heavily branched structure related to having about 5% α-(1,6) linkages [5]. Starch synthesis is hosted in amyloplasts and catalyzed with granular-bound starch synthase (GBSS), specialized for elongation of growing α−1,4 linkage with exclusive or nearly exclusive activity in the soluble phase, and both starch branching enzyme (SBE) and starch synthases (SSs) to construct the branched chains [6]. The glycosyl moieties required for both amylose and amylopectin are provided as ADP glucose synthesized under catalysis of ADP glucose pyrophosphorylase (AGPase) [7,8]. The increased rate of starch biosynthesis during the early stages of tuber induction and the subsequent developmental stages of tuberization is underlined

Starch is an agriculturally important product with multiple food and nonfood valuable purposes. In human nourishment, starch plays an essential role in offering the metabolic energy necessary to perform biological functions; it is the primary source of energy for an enormous portion of the world’s population. As an industrial material, starch has many applications including adhesion, coating, encapsulation, gelling and thickening [5]. Thus, starch content is one of the major quality criteria targeted by potato breeding programs [9] especially in this era characterized by suffering from climate change and growing gap in food supply. 

Traditional potato breeding is a laborious duty due to the tetraploid nature and immense heterozygosity of the potato genomes. The former is the reason for intra-species incompatibilities and inbreeding depression, whereas the latter inhibits the introduction of novel traits through traditional breeding [10]. In addition, screening for functional genetic variation in wild relatives is slow and strenuous [11,12]. Adding to these difficulties, genetic diversity which is the raw material of breeding programs is hindered with vegetative propagation [13] applied for potato cultivation in most countries [14]. 

Based on genetic engineering and in vitro screening, biotechnology can overcome many of potato breeding obstacles [15,16]. However, the health and environmental issues about genetically modified foods [17] put in vitro screening as the first runner of biotechnological tools utilized in potato breeding. Generally, it depends on the genetic variations that arise during in vitro conditions, and these genetic variations are termed somaclonal variations [18]. Such variations can be attributed to point mutations, numerical and structural chromosomal variations as well as epigenetic variations including hypo- and hypermethylation of DNA [19]. Nevertheless, the molecular mechanisms underlying somaclonal variation require further elucidation [20].

In vitro screening of somaclones was successfully used to produce new potato lines with improved tolerance against drought [21], salinity [22], cadmium [15], early blight [23] and postharvest diseases [24]. In addition, several research groups introduced in vitro selected potato lines with improved starch content [25,26,27,28]. However, the genetic expression profile underlining the enhanced starch accumulation was not investigated. The transcription level of several genes can be rapidly and accurately estimated in the same extract using few chemicals, compared with assaying of the corresponding enzymes activities that may require several extraction methods and an arsenal of reagents. The increased rate of starch biosynthesis during the early stages of tuber induction and the subsequent developmental stages of tuberization is underlined with up-regulation of starch synthesis genes that was more pronounced when final tuber size was attained [29].

Generally, in vitro procedures expose plant material to oxidative stress and subsequent mutations [30]. However, although regeneration from preformed meristems (e.g., buds) does not normally produce variants, passing through a callus phase promotes somaclonal variation [31].

Therefore, the aim of the present investigation is to exploit somaclonal variation in callus-sourced regenerated plants in order to select new potato lines with a high starch content and to elucidate the gene expression profiles of starch-related genes, namely, *AGPase, GBSS I, SBE I, SBE II, SS II* and *SS III*.

## 2. Results

### 2.1. Tissue Culture, Acclimatization and Minituber Production

**Figure 1:**
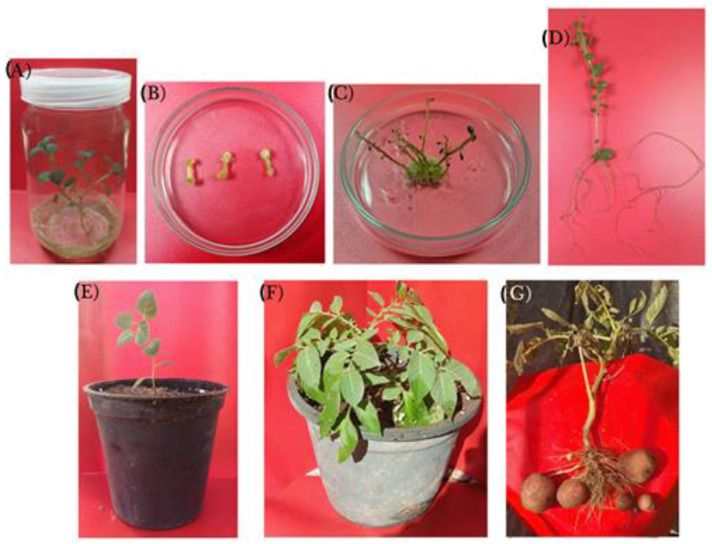
Tissue culture and minituber formation of potato (*Solanum tuberosum*): four-week-old in vitro plants multiplicated via nodal cuttings on basal medium (**A**), callus induction on internode cuttings placed on basal medium fortified with 1-naphthaleneacetic acid (NAA) at 0.186 mg L^−1^ and 6-benzylaminopurine (BAP) at 2.25 mg L^−1^ (**B**), shoot regeneration from calli on basal medium containing BAP at 2.25 mg L^−1^ (**C**), rooting of regenerated shoots on basal medium supplemented with 0.1 mg L^−1^ indole-3-butyric acid (IBA) and 0.5 mg L^−1^ indole-3-acetic acid (IAA) (**D**), acclimation of regenerated plants (**E**), four-week-old acclimated plants (**F**) and minituber formation of acclimated plants (**G**).

Potato in vitro plants (Figure 1A) were selected to provide internode explants. As a response to a callus induction medium, the internode explants swelled with a synchronized appearance of green nodular calli at the cut edges after a couple of weeks (Figure 1B). After being transferred to a shoot regeneration medium, the calli expanded to the whole explant surface.

After eight weeks on the regeneration medium, an average of six shoots per explant (Figure 1C) were regenerated on about 60% of the explants, yielding 146 shoots, of which only 113 (77.3%) were able to survive following three successive nodal-cutting-based multiplication steps and produced corresponding clones. The obtained shoots were rooted successfully on a rooting medium to produce plants ready for acclimatization (Figure 1D). The rooted shoots of all the clones were acclimatized in 5 cm pots (Figure 1E) for two weeks in a greenhouse, and then they were transferred to 25 cm pots (Figure 1F). Fourteen weeks later, the produced G_0_ minitubers were collected (Figure 1G).

The in vitro plants, used as a source of explants, were multiplied via nodal cuttings to establish control clones, and they were designated as meristem-derived (M-D) clone plants. Each of the *M-D* clone plants produced an average of five minitubers, weighing about 25 g. For the callus-sourced clones, a wide spectrum of tuberization potentials was recorded among the different clones. The variation among the different putative somaclones ranged from 2 to 15 for the number of minitubers per plant and 4–27 g for the fresh weight of tubers per plant.

### 2.2. Tuberization and Starch Accumulation

**Figure 2:**
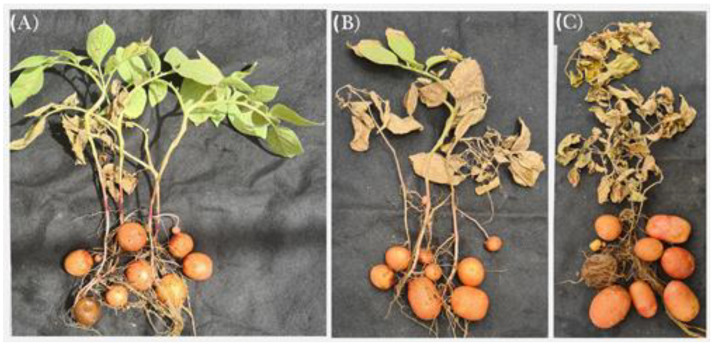
Potato plants carrying G_2_ tubers: the commercial cultivar Lady Rosetta (**A**), *M-D* clone (**B**) and *Ros 119* clone (**C**).

**Figure 3:**
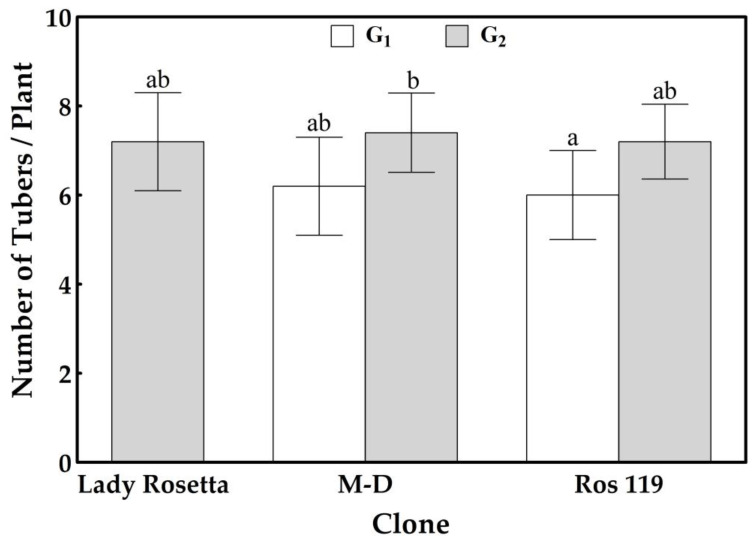
Number of tubers formed by the commercial cultivar Lady Rosetta, *M-D* clone and *Ros 119* clone. Values are presented as mean ± SD of five replicates; bars with different letters are significantly different, based on the LSD test, at *p* < 0.05.

**Figure 4:**
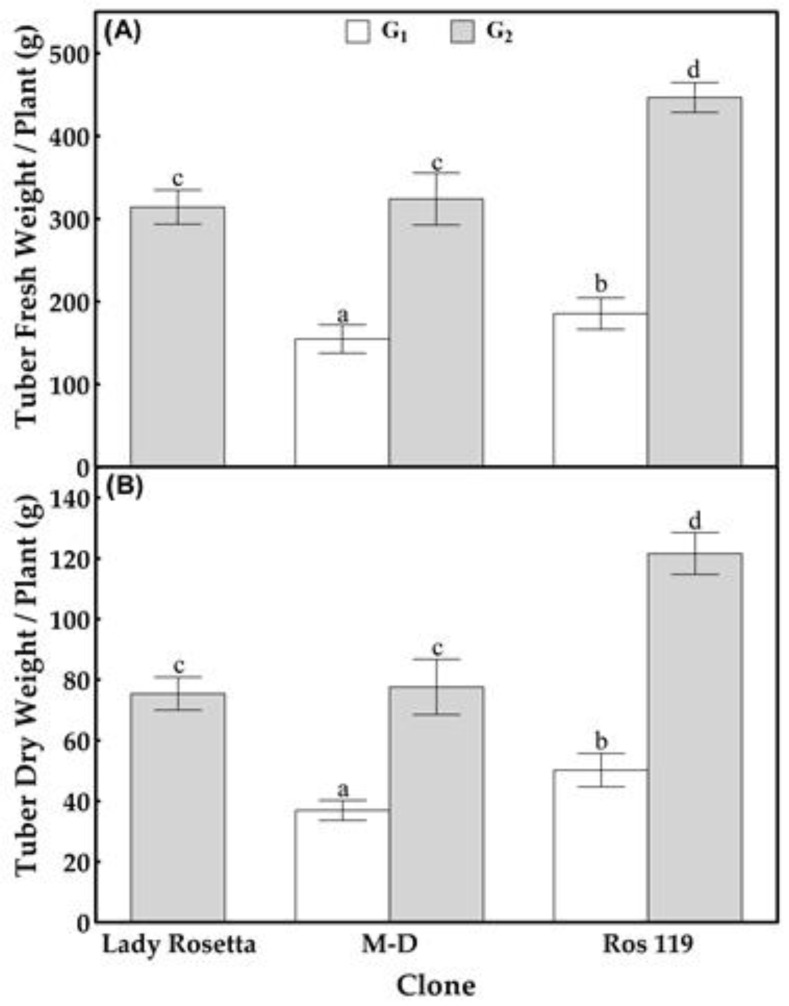
Fresh (**A**) and dry (**B**) weights of tubers formed by the commercial cultivar Lady Rosetta, *M-D* clone and *Ros 119* clone. Values are presented as mean ± SD of five replicates; bars with different letters are significantly different, based on the LSD test, at *p* < 0.05.

**Figure 5:**
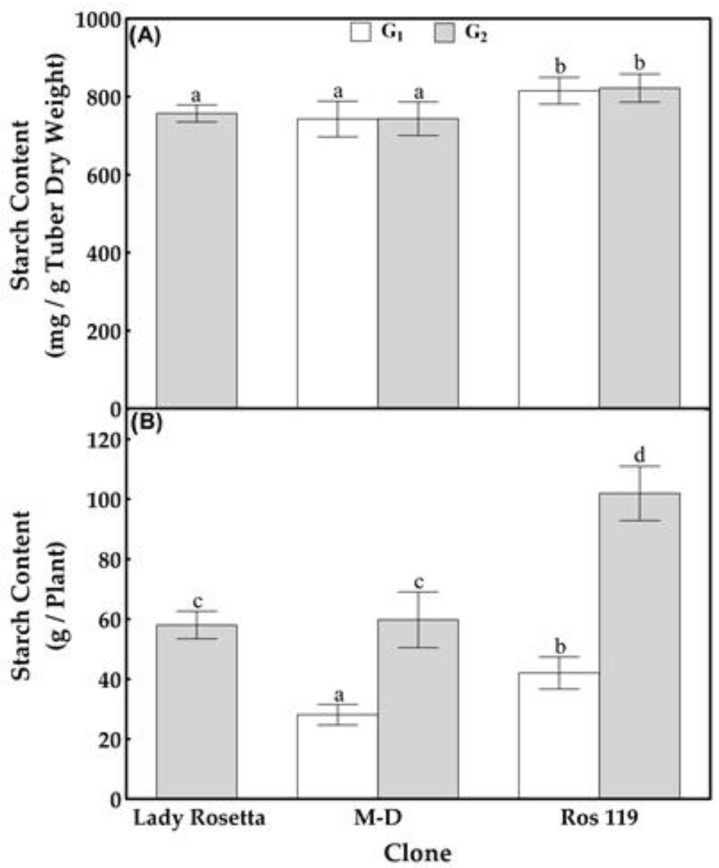
Tuber starch content on dry-weight (**A**) and per-plant (**B**) bases in tubers formed by the commercial cultivar Lady Rosetta, *M-D* clone and *Ros 119* clone. Values are presented as mean ± SD of five replicates; bars with different letters are significantly different, based on the LSD test, at *p* < 0.05.

Compared with the G_1_ tubers produced by the *M-D* clone, the primary screening of the G_1_ tubers produced by the 105 callus-sourced clones failed to achieve better tuberization potentials in terms of the number of tubers, tuber weight and starch content, except for the clone named *Ros 119*. Although bearing the same number of tubers produced by the *M-D* clone (Figure 2 and Figure 3), *Ros 119* exhibited tubers with 20 and 36% higher fresh and dry weights (Figure 4), in addition to 10 and 49% increases in starch content on the bases of tuber dry weight and average content per plant, respectively (Figure 5). The previous superiority of the *Ros 119* clone over the *M-D* one was observed to be intensified in the G_2_ tubers. Compared with the *M-D* clone, the *Ros 119* tubers exhibited 38, 57, 11 and 71% increases in fresh weight, dry weight, starch content expressed in mg/g dry weight and starch content expressed in g per plant, respectively.

Based on the results of the present study, the cv Lady Rosetta produced an average of 7.2 tubers per plant (Figure 2 and Figure 3), weighing 314 and 75 g on the bases of fresh and dry weights, respectively (Figure 4). The starch determination in the tubers reflected their ability to accumulate about 756 mg of starch per g of dry weight, resulting in a total starch content of about 58 g per plant (Figure 5). Similar results were recorded when observing the G2 tubers produced by the M-D clone.

The primary data for the average weights of the G_2_ tubers produced by the callus-sourced clones introduced *Ros 119* as a distinguished clone possessing fresh and dry weights that were about 42 and 61% higher, respectively, than those of the Lady Rosetta tubers. Similarly, the screening for starch accumulators among the callus-sourced clones reflected the ability of *Ros 119* to accumulate a starch content that was 10% higher (Figure 5Athan that of the cv Lady Rosetta tubers, without an accompanied significant variation in the average number of tubers per plant. The combined increases in tuber dry weight and starch content in *Ros 119* result in about a 75% increase in starch content per plant (Figure 5B).

### 2.3. Gene Expression Analysis

**Figure 6:**
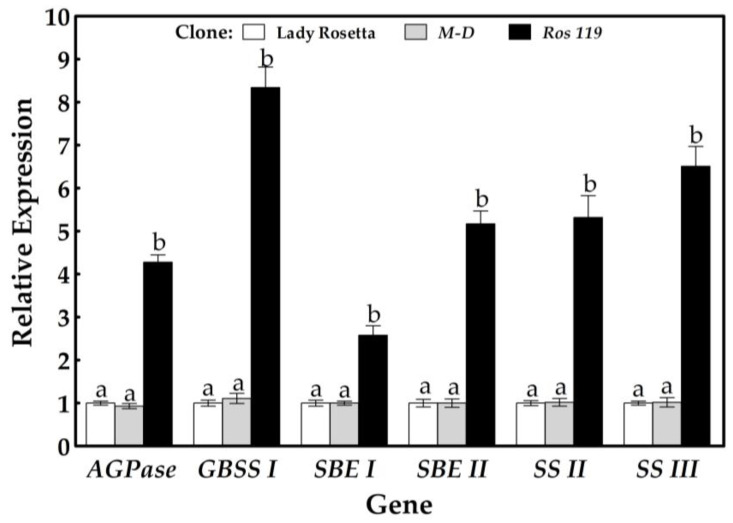
Relative expressions of starch-synthesis-related genes in tubers formed by the commercial cultivar Lady Rosetta, *M-D* clone and *Ros 119* clone. Values are presented as mean ± SD of five replicates; bars with different letters are significantly different, based on the LSD test, at *p* < 0.05.

The monitoring of the gene expressions of the key enzymes involved in starch synthesis reflected an insignificant difference between the expression levels in the Lady Rosetta tubers and the corresponding gene expressions in the *M-D* clone tubers (Figure 6). However, the callus-derived clone *Ros 119* exhibited significantly elevated transcript abundances for the examined genes, compared with the corresponding genes in the Lady Rosetta tubers.

In *Ros 119*, the expression level of the ADP glucose pyrophosphorylase (*AGPase*) gene responsible for the key reaction in starch content reached a 4.29-fold increase compared to the level quantified in the Lady Rosetta tuber. The transcript abundance of the granular-bound starch synthase (*GBSS*) gene responsible for amylose biosynthesis exhibited the most obvious enhancement among the examined genes, manifesting a 8.34-fold increase compared to the corresponding gene expression in the Lady Rosetta tubers. The expressions of the starch-branching enzyme (*SBE*) genes responsible for α−1.6 linkages in amylopectin exhibited 2.57- and 5.17-fold increases compared to the expressions measured in the Lady Rosetta tubers for *SBE I* and *SBE II*, respectively, while the expressions of the starch synthase II (*SS II*) and starch synthase III (*SS III*) genes responsible for α−1.4 linkages in the branched chains reached 5.31- and 6.5-fold increases, respectively, compared to the corresponding gene expressions.

## 3. Discussion

In the current study, callus was induced on internode explants on a medium supplemented with 2.25 mg L^−1^ BAP and 0.186 mg L^−1^ NAA, while shoot regeneration was achieved following auxin removal and the addition of AgNO_3_ at 4 mg L^−1^. The same media were utilized for callus induction followed by shoot regeneration from potato leaves and internode explants in a previous study [32]. Similarly, Kumlay and Ercisli [33] employed a medium supplemented with both cytokinin and auxin to induce callus formation on the leaves and internode explants of four potato cultivars, whereas shoot regeneration commenced following auxin omission. The same approach was implemented by Ghosh et al. [34], who started with leaf-sourced explants of three potato cultivars.

Auxins are important players in callus initiation [35]; they afford a narrow range for cell fate transition [36], which necessitates their withdrawal or at least the lowering of their concentration in regeneration media. However, cytokinins influence callus initiation [33]; they are the major participants in regeneration media, where shoot regeneration is the consequence of interconnections among cytokinin receptors, the development of shoot meristems and cell cycles [37]. The role played by AgNO_3_ in shoot regeneration media can be attributed to the intrusion with ethylene perception, which mitigates the hindering influence of the accretion of the gas hormone on shoot regeneration [38,39]. In addition, AgNO_3_ enhances the accumulation of polyamines [32], whose role in the improvement of morphogenesis has been previously recorded in potato shoot cultures [40].

In the present study, the rooting of the regenerated roots was achieved on a medium supplemented with 0.1 mg L^−1^ IBA and 0.5 mg L^−1^ IAA. A combination of the aforementioned auxins was utilized by Hajare et al. to initiate the rooting of potato-regenerated shoots [41]. IBA is generally employed to root potato-regenerated shoots, either alone [42,43] or in combination with another auxin [44,45].

The genetic variations that regularly arise during regeneration from callus cells are attributed to numerical and structural chromosomal variations; point mutations; and epigenetic variations, including the hyper- and hypo-methylation of DNA [19]. These variations, termed somaclonal variations [18], introduced *Ros 119* as a new clone. *Ros 119* is a starch-rich clone able to accumulate a significantly higher amount of starch in its tubers compared with the commercial cultivar Lady Rosetta. Similar to our results, Thieme and Griess [27], Rosenberg et al. [25] and Bayati et al. [24] used somaclonal variation to introduce potato lines with enhanced starch content. However, the introduced lines had different starch accumulation potentials and different frequencies in their occurrence, which could be attributed to the randomness of the variations responsible for the enhanced starch accumulation.

**Figure 7:**
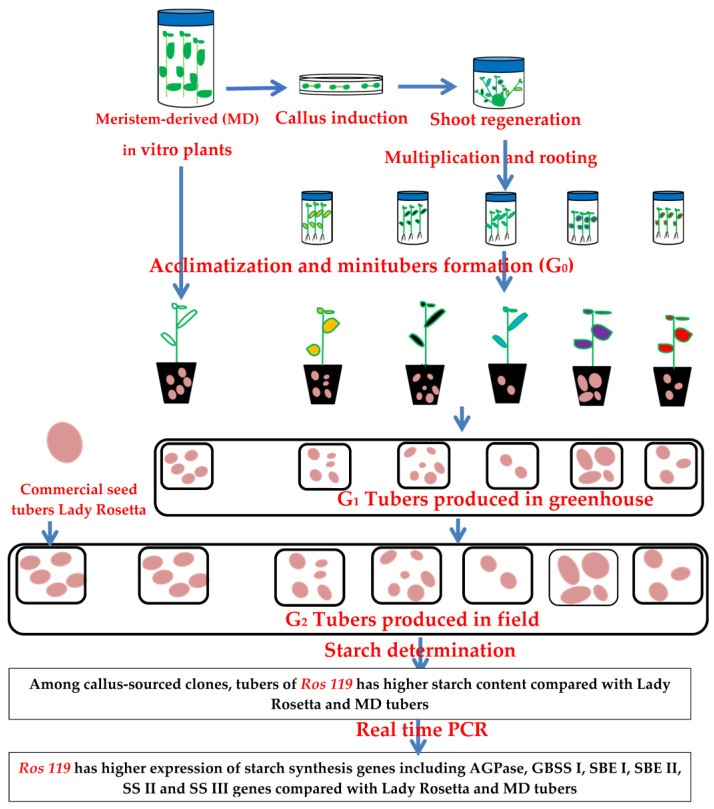
Graphical summary showing the production of potato somaclones with distinguished ability to accumulate starch based on upregulation of starch-synthesis-related genes.

The increase in the starch accumulation recorded in the present study can be attributed to the increase in the expression of AGPase, SSs, GBSS and SBEs genes (Figure 7). The up-regulation of starch synthesis-related genes was documented during tuber formation of potato [29,46]. Based on microarray analysis, Kloosterman et al. [46] recorded up-regulated profile for the genes addressed in the present study during the early stages of tuberization, that was maintained until the final tuber size was reached. The authors attributed these results to the increased rate of starch biosynthesis. Supported with results of transcriptome analysis, Ferreira et al. [29] documented low expressions of starch-biosynthesis-related genes at early tuberization stages followed by upregulation at terminal stages.

Variations in gene expressions in tissue-culture-derived plants have also been documented in *Phalaenopsis* ‘Wedding Promenade’ [46] and rice [47]. When investigating the fruit transcriptome of cucumber somaclones, Pawe\lkowicz et al. [48] documented a different differential gene expression in each clone. The authors attributed the results to variations in the genic region and the interactions among molecular networks, which initiate specific pathways. Similar findings were recorded by López-Hernández and Cortés [49], who examined the transcriptomes of mint somaclones.

An increase in starch content accompanied by an increase in the expression of the *AGPase* gene was previously recorded in potato by Müller-Röber et al. [50,51] and Stark et al. [50,51]. The synchronization between the increase in starch content and the expressions of the *AGPase, SS, GBSS* and *SBE* genes has been recorded in potato [8], rice [52], wheat [53] and lanzhou lily bulb [54].

AGPase catalyzes the key step in starch biosynthesis; it produces ADP glucose, which provides the glycosyl moiety required for starch biosynthesis [7,8]. Thus, AGPase is the rate-limiting enzyme in starch accumulation in potato [55]. ADP-glucose is actively transferred through the activity of specialized transporters into amyloplasts [56,57] for the subsequent synthesis of amylose and amylopectin. Starch biosynthesis is catalyzed by a group of enzymes, including GBSS, SSs and SBEs. Both GBSS and SSs are responsible for chain elongation by catalyzing the formation α−1,4 glucosidic bonds in amylose and amylopectin, respectively, while SBEs are responsible for the formation of α−1.6 linkages at the branch points of amylopectin [6]. Several SSs have been characterized in plants; however, they are mainly referred to as SS II and SS III in potato [8].

## 4. Materials and Methods

### 4.1. Explant Preparation

Virus-free tubers of potato (*Solanum tuberosum*) cv Lady Rosetta were kindly provided by the Agricultural Research Center, Cairo, Egypt. The tubers were kept at 10 °C until sprouting; then, the sprouts were separated from the tubers and immersed in tap water mixed with a few drops of a commercial liquid detergent, agitated for 10 min and then thoroughly rinsed in running water for 30 min to remove the remaining detergent. The washed sprouts were placed in a 250 mL caped jar containing about 100 mL of 20% commercial Clorox (5% chlorine) (Clorox Egypt) containing a few drops of Tween 20, and then the jar was shaken for five minutes. In a laminar flow cabinet, the surface-sterilized sprouts were picked and rinsed thoroughly in sterile distilled water. The meristems were aseptically excised and transferred into sterile tubes (one explant/tube), each containing 10 mL sterile basal medium (MS medium [58] supplemented with 100 mg L^−1^ myoinositol and 30 g L^−1^ sucrose) supplemented with 0.01 mg L^−1^ NAA, 0.1 mg L^−1^ gibberellic acid (GA_3_) and 2 mg L^−1^ calcium pantothenate. The medium was solidified using 7 g L^−1^ agar, and the pH was adjusted to 5.7 before autoclaving for 20 min at 121 °C. The cultures were incubated at 25 °C and at a light intensity of 15.8 Wat m^−2^ with 16/8 h light/dark cycles under cool white fluorescent lamps. The same incubation conditions were implemented throughout the study. After six weeks, shoots that were about 5–7 cm were collected and cut into nodal cuttings for micropropagation in order to establish control clones designated as meristem-derived (*M-D*) clones, which were employed as a source of internode explants for callus initiation. The plant material was subcultured at three-week intervals in 400 mL glass jars (4–5 cuttings/jar), each containing approximately 50 mL basal medium.

### 4.2. Callus Induction, Shoot Regeneration and Rooting

Based on our previous publication [32], about 1 cm of each of the internode explants was utilized for callus induction on a basal medium augmented with 2.25 mg L^−1^ BAP and 0.186 mg L^−1^ NAA. Six weeks later, the explants carrying calli were transferred to a regeneration medium consisting of a basal medium supplemented with 2.25 mg L^−1^ BAP and 4 mg L^−1^ AgNO_3_. After eight weeks, the regenerated shoots were separated from the remaining callus and each subjected to three cycles of subculture on a basal medium in order to produce a sufficient number of shoots considered as a putative clone that received a characteristic code. The shoots of each clone were transferred to a rooting medium consisting of a basal medium supplemented with 0.1 mg L^−1^ IBA and 0.5 mg L^−1^ IAA [41]. Four weeks later, the healthy rooted plants were harvested and acclimatized.

At the beginning of September 2019, the healthy rooted shoots of all regenerated clones, including those of the *M-D* clone plants, were carefully collected and rinsed thoroughly with tap water to remove the remaining culture media adhering to the roots. The washed plants were transplanted into 5 cm pots (one plant/pot) filled with an autoclaved soil mixture of sand: peat moss: vermiculite (1:1:1, *v*/*v*). The plants were covered with transparent plastic bags to retain a high humidity, irrigated regularly with sterilized water and maintained in a greenhouse. Two weeks later, the plastic bags were punctured with a paper punch and kept for another week; thereafter, the acclimated plantlets were transplanted in 25 cm pots. At the end of December, G_0_ minitubers were harvested, washed and stored at 2 °C until they were used as seed tubers.

In the middle of February 2020, the G_0_ minitubers of each clone were planted in 1 × 1 × 0.25 m boxes containing peat moss:sand:vermiculite:perlite:foam (40:40:10:5:5) in a greenhouse following the recommendations of the Egyptian Ministry of Agriculture for agricultural practices concerning cultivation, fertilization, irrigation and pest and disease control. After 16 weeks, the G_1_ tubers of each clone were harvested, washed, counted and weighed. The tuber samples of each clone were dried at 50 °C until constant weight and used for starch quantification, while the remaining tubers were stored as seed tubers at 4 °C. At the beginning of September 2020, the stored G_1_ tubers were planted in an open field for field experiments, and G_2_ tubers were collected 16 weeks later. The field experiments were carried out in Samannud, Gharbiya Governorate, Egypt. (30°52′54″ N 31°14′11″ E). The soil at the study area was loamy soil with pH 7.8 and an electrical conductivity (EC) of 2.96 EC/dsm^−1^. The research site’s weather was semi-arid with rainy winter (rainfall of 10 mm, average day/night temperature of 19/10 °C and relative humidity of 60%). Seven tubers of each clone were planted at a depth of 20 cm, with an in-row spacing of 25 cm and an inter-row spacing of 72 cm. The agricultural practices guidelines of the Egyptian Ministry of Agriculture were followed for cultivation, fertilization, irrigation and pest and disease control. The tubers of the commercial cultivar Lady Rosetta were planted under conditions the same as those of the control clones. After 16 weeks, five plants of each clone were randomly picked for sampling. The tubers of each plant were counted and manipulated separately, where they were washed with water to remove any remaining soil particles and dried with a clean towel. After the determination of the total fresh weight of the tubers of each plant, three sections of about 5 mm thickness from the bottom, middle and top of each tuber were collected using a sharp knife. The collected sections from the tubers of each plant were mixed and further cut into fine pieces before weighing out one g to be stored at −80 °C in order to represent the plant in a qPCR analysis. The remaining pieces were added to bulk tuber parts and dried at 50 °C until constant weight for dry weight determination and starch quantification.

### 4.3. Starch Determination

The sugar-free dry tuber tissue prepared via the repeated extraction in iso-propanol (80% *v*/*v*) was used for starch quantification. After an overnight drying step at 70 °C as described by Kumar et al. [59], the dried fragments were homogenized in perchloric acid (60% *v*/*v*) for starch hydrolysis. The liberated glucose was estimated using the anthrone method [60].

### 4.4. Real-Time Quantitative PCR

The stored tuber samples were crushed into a fine powder in liquid nitrogen and used for total RNA extraction using an RNeasy Plant Mini kit (Qiagen, Hilden, Germany), with the purification step using DNase according to the manufacturer’s instructions. RNA purity and concentration were confirmed with a NanoDrop spectrophotometer (ND 2000c, Thermo Fisher Scientific, Wilmington, DE, USA). One μg of the purified RNA was converted to cDNA using a Sensi-FAST™ cDNA synthesis kit following the standard protocol from the manufacturer. Quantitative real-time PCR for AGPase, GBSS I, SBE I, SBE II, SS II and SS III cDNAs was carried out on a Mx3000P (Stratagene, CA, USA) qPCR system using specific primers (Table 1) [8]. The thermal profile of the real-time system was 95 °C for 10 min, followed by 40 cycles (95 °C for 15 s and 60 °C for 60 s).

The relative transcript abundance of tublin was used as an endogenous control, to which the transcription levels of the AGPase, GBSS I, SBE I, SBE II, SS II and SS III genes were normalized using the 2^−DDCt^ method [61]. The expression recorded in the tubers of the commercial cultivar Lady Rosetta was employed as a quantification unit.

### 4.5. Statistical Analysis

The results of all clones are presented as the mean of five replicates ± standard deviation (SD). One-way ANOVA with the LSD post hoc statistical test was utilized to compare the different clones on the basis of tuber yield per plant manifested in number of tubers, the fresh and dry weights of the tubers, the starch content on dry-weight and per-plant bases and the expressions of six starch-related genes at *p* = 0.05 using SPSS v. 14.

## 5. Conclusions

Somaclonal variation is an efficient breeding tool. It can provide breeders with new genotypes with favorable traits, which is essential for breeding programs targeting yield improvements. Somaclonal variation is a time-efficient alternative to conventional breeding, and it is able to provide potato clones with an outstanding potential for starch accumulation at a reasonable cost. It is an acceptable approach that is able to manipulate starch synthesis genes without issues arising regarding genetic transformation. However, the emerging clone requires genetic characterization to participate effectively in breeding programs targeting qualitative and quantitative yield improvements, which is the aim of our future work.

## Figures and Tables

**Table 1:** List of primers used for qRT-PCR (5′–3′).

Gene	Identification ID	Primer Sequence
*Tublin*	LOC102577624	5′-GTCAGTCTGGTGCTGGTAATAA-3′ 5′-TCTCAGCCTCCTTCCTTACA-3′
*AGPase*	LOC102577790	5′-TT CCTT CCACCAACCAAGATAG-3′ 5′-CACTATGG AGTGTT CCACAGAA-3′
*GBSS I*	LOC102583115	5′-CTTGCGTTTGCTGAGATGATAAA-3′ 5′-CAGAAGCTCCTAAGCCCAATAG-3′
*SBE I*	LOC102596498	5′-GCGAACATGTGTGGCTTATTAC-3′ 5′-TCTCGTCACTCTCCTCGATATT-3′
*SBE II*	LOC102590711	5′-CTCTGGATAGACCGTCAACATC-3′ 5′-AGGTACCCTT CTCCTCCTAATC-3′
*SS II*	LOC102583115	5′-CAACAGGACCTACTTCAACAGA-3′ 5′-CTACCACTCCCACCATCATAAG-3′
*SS III*	LOC102577674	5′-GTCACCTGTTCGTGTATCATCT-3′ 5′-CCACTCTCTT CCGATCTCTTTG-3′
